# Firsts in their field: the perceptions of women who have led the way

**DOI:** 10.3389/fspor.2024.1417406

**Published:** 2024-12-19

**Authors:** Margaret Stone, Pippa Chapman, Urvi Khasnis, David Collins

**Affiliations:** ^1^ East Tennessee State University, Center of Excellence for Sport Science and Coach Education, Johnson City, TN, United States; ^2^ School of Education and Sport, University of Edinburgh, Edinburgh, Scotland, United Kingdom; ^3^ School of Health and Human Performance, Dublin City University, Dublin, Ireland

**Keywords:** leadership, barriers, coping, challenges, attributes

## Abstract

The role women play in sport has been the subject of considerable discussion and research since the nineteen seventies. Much of this research has been descriptive or focused on the barriers, women face in advancing into a leadership role in a sporting organization. In an attempt to enrich the picture, women role models who occupy or have occupied leadership roles in sporting organizations were identified to gain their perspective, in their own words, on the challenges and requirements needed to be successful. The experience of these high achieving women has value in paving the way for future generations with ambitions to succeed in a leadership role in sport. Six women were identified and interviewed as the first to occupy their position in a sporting organization. Using Interpretive Phenomenological Analysis data were thematically analyzed against three predetermined themes, attributes they possessed, challenges they faced, and coping skills they used in aiding them to succeed in their positions.

## Introduction

Sport has in the past been seen as a male dominated environment. Justifiably and increasingly challenged, the drive to increase the number of competitive opportunities for women are now paralleled by calls to increase the number of women involved in the leadership and management of sport at all levels (1). Despite a trend towards change, however, female representation in leadership roles of sporting organization still remains minimal (2). The International Olympic Committee (IOC) reports a small growth but still disproportionate balance in the percentage of women coaching at recent Olympic Games (IOC 2022—Rio 2016—11%, Tokyo 2020—13%.). Within the US system, Acosta and Carpenter's (3) 25-year longitudinal study of women participating in intercollegiate athletic programs in administrative and coaching roles offers an even more negative picture. Whilst more than 90% of women's teams were coached by women after the historic Title IX legislation was applied in 1972; the numbers went down to 45.6% in 2000 and a further reduction to 44% by 2002 (3).

The picture is even more disproportionate in sports leadership. Only 18% of women's programs are directed by females, whilst almost 20% of female team structures have no females (4). Although their excellent and ongoing research does not offer any figures for male sports, one can only assume that the balance is even more one-sided. In short, females in sports leadership and management are a rare breed, with no particular signs of change on the immediate horizon!

Positively, there are initiatives targeting this issue; for example, the WISH project has a goal of increasing the number of women coaching at the Olympic level (5). Furthermore, the exact reasons for the challenges facing aspiring women administrators or coaches has been the topic of considerable discussion (6, 7). Although positive and clearly well-intentioned, however, much of the current literature is somewhat descriptive (6, 8–10), and can often lack the action focus required to drive a more balanced and appropriate workforce.

Other literature does hold the potential to drive action, although much of this work is focused more on the negative “blockers” which are perceived to limit progress. It has been found that women are often excluded from networking opportunities, especially at the higher- level of organizations (11). A lack of mentorship has also been highlighted (12). Speculation regarding gender bias (13) and homologous reproduction (14) are further reasons presented for the lack of female leadership. Reported minority stress and incidents of discrimination (15) are also common. Even recent literature, whilst stressing the twin track, individual *and* organizational approaches needed, may offer too little to the hard pressed but ambitious female wishing to climb the ladder (16). Finally, the lack of numbers, and consequent social support (17), are also presented as limiting progress.

These sources provide descriptive information about the number of women involved in high performance sport, and usually highlight the problems women face rather than considering their experiences more holistically. While it is important to understand that women struggle to progress into decision- making positions or assume leadership roles in sporting organizations (18), however, it is also important to offer real world role models. In short, rather than documenting the situation or describing the issues women face, a prudent additional approach might be to ask women who made it for *their* perspective.

Therefore, the aim of this study was to solicit the perspective of women who were first to occupy their role and have been or are presently in high performance positions. Further, because much of the research has focused on barriers or problems faced by women, it is essential to change that focus and gain their views, in their own words, as to the requirements and challenges of such a position and their pathways to achieving decision making roles in sports.

## Methods

For the study, Interpretative Phenomenological Analysis (IPA) (19) was used to explore the “lived experiences” of the participants. The participants were selected through personal contacts of the lead researcher based on purposive sampling, a valuable non probability sampling technique (20) through which participants are identified from a population based on the experience and knowledge of the researcher. In accordance with IPA recommendations, semi-structured one-on-one interviews were chosen as the method of data collection. Finally, approval to conduct this study was given through the Ethics Committee at the University of Edinburgh.

### Participants

Inclusion criteria required each participant to have extensive experience in either a technical and/or leadership position for a minimum of fifteen years and have been the first female to hold such a role in their sport organization. We asked participants if they could be named in order to add to the validity of the study. The final sample consisted of three participants from sport administration roles, two coaches and one athletic trainer, ranging in age from fifty-one to seventy-one years old. Names and primary roles are shown in Table 1.

**Table 1:** Participants.

Name	High status achievement/role
Travis Triplett	1st. Female President of the U.S. National Strength and Conditioning Association.
Sue Hillman	1st. Female Athletic Trainer in the National Football League
Rocky La Rose	1st. Female Athletic Director University of Arizona
Andrea Hudy	1st Female strength coach -University of Kansas National Men's Basketball Championships (NCAA).
Ursula Papandrea	1st. Female President of the International Weightlifting Federation
Teena Murray	1st. Female Vice President of Health and Performance for the Sacramento Kings Professional Basketball Team (NBA)

### Procedure

Each of these women were contacted by email and telephone initially to determine their interest in participating in this study, and all six agreed. Once confirmation of their participation was received, a Participant Information Sheet (PIS) and the informed consent documents were sent to them. It was stated on the PIS that the participants would be named as this would strengthen the credibility and authenticity of the study. Additionally, they were informed they would be given two opportunities to review their statements: firstly, in the transcripts and secondly in the finished paper pre-publication and they could edit as they felt necessary. An interview timeline was established via email and telephone.

At the design stage, questions were developed with three predetermined themes or concepts; namely, the participants attributes, challenges, and coping mechanism dealing with challenges (21). A pilot interview lasting sixty minutes was scheduled with a female former sports organization administrator to refine the researcher's interviewing technique and questions. After the pilot interview no questions were changed.

Initially, participants were asked for a description of their current role or role they occupied as a first in the field. This allowed for rapport to develop between the participant and researcher before the conversation turned to the key research questions. Firstly, attributes or character traits they believed they exhibited leading them to occupy the position they held. Secondly, the challenges they faced in their professional life, and thirdly the coping mechanisms used while faced with those challenges. Finally, they were asked for overall reflections on their careers. The interviews were semi-structured by design and open ended allowing for flexibility in responses (19). Probes were also used when necessary to expand on areas of particular interest. The interviews were conducted and recorded on Microsoft Teams and the transcripts retained for analysis with the permission of the participants. During the interviews, notes were taken by the interviewer to record any notable information which could be useful during analysis. During these interviews, the information covered moved from the more general to more specific details as the subjects became more comfortable with the process and recalled events they had experienced.

### Member reflections

To ensure the trustworthiness and internal validity of the study, transcripts and identified quotations were emailed to the participants to confirm the accuracy of the statements and information imparted during the interview process. This form of member checking is a useful means of confirming credibility of the findings (22).

### Data analysis process

The six interviews were transcribed verbatim. The interview durations ranged from thirty to sixty-seven minutes with an average duration of forty-nine minutes. Data analysis was based on IPA which has a specific theoretical background in phenomenology. This approach concentrates on the “lived experiences” of the interviewed individuals. It is also hermeneutic in that it relies on the researcher's interpretation of these lived experiences (23). Furthermore, IPA allows for exploration of an individual's personal perception or account of events, or state of being, as opposed to attempting to produce an objective record of an event. Lastly, IPA is also a strongly idiographic approach concerned with a detailed analysis of one case either as an end, or before moving to similarly detailed analysis of other cases, and therefore, is an ideal form of analysis for this study (19).

Following the recommendations of Braun and Clarke (24), the initial steps of analysis focused on data familiarization including extensive reading and re-reading of the raw data. During the reading process notes were taken by the researcher. Themes and further sub-themes developed through the process were consistently re-checked with the transcripts to ensure they were indeed confirmed by the primary source, the participants' own words. Themes had been identified while developing the questions for the interviews, they are an intricate part of qualitative research. Themes cannot be observed as they are in the form of perceptions and emotions experienced in the mind of the participants, and as such are at the heart of the participant's story (24). These pre-identified themes of attributes, challenges and coping skills were the central concepts around which the participants discussed their “lived experiences” (24). As this process advanced, sections of text were grouped together into 'shared meanings' (Shared Meaning Units or SMU) (25) from the identified sub-themes. Quotations were then identified relating to these shared meanings and examples from the raw data recorded. The predetermined themes were noted as key or central concepts (Central Organizing Concepts; COCs) of the recorded raw data.

## Results

All six participants confirmed that they were satisfied with their transcripts. Further, all participants received a summary of extracts taken from the raw data. The lead researcher emailed these extracts to the six participants requested they review the extracts for accuracy and asked them to edit as they felt appropriate to provide further clarity and depth to the data (26). Five of the six participants chose to provide further input at this stage, with the sixth satisfied with the information provided. The number of participants with shared meanings noted, and quotes from the raw data to illustrate the “shared meanings” produced the results presented in Table 2.

**Table 2:** Summary of themes Sub themes and quotations from the Raw data.

Central organizing concepts (COCs)	Shared meaning units (SMUs)	Raw data codes (*N* participants)	Exemplar quotes
Attributes	Leadership Skills	Developing New Leaders (6)	“My next project, if approved by the board will be to develop a women's leadership pipeline” (Ursula)
		Competitive Culture (6)	“I want a culture of hard competitive work. I create that culture” (Andrea)
	Relationships	People Skills (5)	“People do not quit jobs they quit people.” ((Andrea)
		Proven Team builder (5)	“I think what I had proven was that I knew how to build a team.” (Teena)
Challenges	Being Female	Obvious Identification (6)	“Unfortunately, the first thing somebody sees is your gender” (Andrea)
		Conscience of being a role model (6)	“I was entering a space where women had never been before …I sure didn’t want to screw it up” (Rocky)
	Feeling Supported by mentors/Male advocate	Finding the mentor (6)	“I had some very progressive men I worked with.” (Rocky)
		Male coaches making a difference (6)	“Coach Martin was absolutely wonderful and supportive” (Andrea)
	Non- Supportive experiences	Potential Legal issues (5)	“I believe the treatment I received would have been considered harassment and verbal abuse” (Ursula)
		Non- verbal Communication (5)	“I am not sure that everyone in the building was excited about having a woman in a leadership role” (Teena)
Coping Skills	Self-Belief	No awareness of her impact	“I just did what I did and I didn’t know I was opening any doors” (Sue)
		Having and accomplishing a vision	“I knew how to impart a vision and move the needle towards accomplishing that vision” (Teena)
	Perseverance	Having an impact	“Yes, I had a huge impact, but I could have done more” (Andrea)
		Dealing with situations	“It was a little bit of learning, how to best handle situations” (Travis)
	Confidence	Self –awareness (6)	“I would argue I have one of the strongest resumes in college basketball men or women” (Andrea)
		Altruistic Behavior	“I am here so you can focus 100% on football…so again trying to build trust” (Andrea)

### Attributes/character traits of women firsts

We interviewed six women occupying technical and leadership positions which, until their arrival, had only been occupied by a male. This revealed character traits which might have been expected, such as coping with stereotypical barriers (16), the ability to demonstrate leadership capabilities and the ability to develop working teams. These abilities become more interesting when considering the challenges these women faced during their tenure, and their methods used in coping with those challenges. Teena speaks of her abilities to lead and how she was approached and chosen as the first female to hold the position of Vice President of Health and Performance for the Sacramento Kings, a professional team in the National Basketball Association (NBA),

My title was Vice President of Health and Performance, and it was a new role created by the organization the first time to have a leader charged with bringing the worlds of medical and performance together and trying to implement what we would call a high-performance approach. Which of course means an interdisciplinary player- centric, integrated approach and I was hired by one of the assistant general managers. When I came into the role, I knew it would take time to develop this new approach and to understand what this role was supposed to be.

Teena goes on to emphasis her thoughts on high performance coaching,

It's an area that I'm passionate about so I'm grateful that I've had these opportunities but it's not easy, it's not easy…There is still a lot of work to do, and I do think a transformational leadership style, which is what I've, I've really tried to focus on myself and studied in my own, is important.

She is referring to the development of a high- performance model which sports generally are still struggling to develop (27). Like Teena, Andrea Hudy was involved in Basketball, Andrea was the strength and conditioning coach in Men's Basketball. She was the first female Strength and Conditioning Coach to coach a Men's National Collegiate Athletic Association (NCAA) Championship team at the University of Kansas. In reference to her position, she indicates that a primary aspect is having the ability to build relationships with the players and coaches to accomplish a goal.

What I always fall back on, is anybody that I worked with, hopefully would say we had a good relationship, and I did challenge them and help them not only to be a better athlete but a better person…I think people do not quit jobs they quit people. My work ethic always lies on building a good understanding of the expectation of winning as well as creating good relationships.

Leadership is about building and sustaining relationships (28). Generally, all six of these women exhibited strong leadership abilities; they built relationships which not only benefited them, but also those with whom they were working, and those relationships lasted throughout their career as firsts. Sue Hillman emphasizes how lasting relationships influenced her chosen career path and still play a role presently. At age seventy-two in the 2024 summer season, she was again to assume the role of Head Athletic trainer for the Pittsburgh Maulers, a USFL team, unfortunately the team dissolved before the 2024 season.

The people I worked with tend to lift me up later in life…Rick Burkholder he was my assistant at Arizona he was a graduate student, and I put him on staff, he went to Pittsburgh with the Steelers and when I resigned at Arizona, he called me right away and said come work camp with John and I. So, he told John and together they invited me to camp and that opened the door for other things.

There is no doubt that leadership and relationships are intertwined, there are numerous examples from all six women of how their leadership skills and the relationship they have built serve as hallmarks of not only their careers, but how they have supported others in their endeavors.

### Challenges

Although these women have all been successful in their chosen careers, it is of interest to explore the challenges they have faced along the way. Travis addresses the issue of being a female in a male dominated sports organization. “Being a female there's probably going to be a little more scrutiny…when the majority of the organizational membership are men. So, I didn't dwell on it or worry about it.” Likewise, Rocky was conscious of being the first woman at her university to play a supervisory role over men's American Football and men's Basketball. She gave considerable thought to her approach to the role,

Believe it or not, I put a word on a piece of paper the word was infiltrate, and that was what I attempted to do. I knew domination was not going to work. I knew saying I was the boss wasn't going to work and, I know I didn't maybe know enough at the time to pull it off …I knew I was going to do things a little differently than my previous male colleagues.

Rocky's considered approach to the role, set her up for longevity in her position. Several of the participants expressed gratitude to male mentors, who then became a supportive facilitator recommending them for the “first's” position. Sue speaks to this support when she refers to John and Rick and the support she was given in the NFL, and again Rocky refers to the coaches she worked with in supervisory capacity,

I had some very progressive men I worked with and one of them felt it was important to get women involved in Athletics… one of them put me overseeing the day-to-day operations of Football and Men's Basketball the two high power revenue coaches that I was working with… am grateful to all three of them to be honest.

Although several of these women discussed gratitude for the opportunities that male colleagues presented to them, there were also challenges these women faced that should be considered non- supportive. Ursula speaks to the challenges she had with male colleagues when she assumed the position of President of the International Weightlifting Federation.

The obstacles came April and May of 2020 when I was in a leadership position…and I mean combative like in any other arena there would have been; all sorts of harassment and verbal abuse…if I had been a man, I would not have been treated that way.

Ursula's work involved close professional relationships with Eastern European men who had dominated the International Weightlifting Federation for decades. It is interesting to note the cultural differences she experienced, unlike Andrea whose work experience was focused on the American sport of Men's Basketball but states clearly, she faced a challenge of a different kind but a challenge, nevertheless,

I would argue that I have one of the strongest resumes in college Basketball both men or women and people don't look at me to fill a position because they first see I am a woman and I know that for a fact. I did not see it back then but looking back on it now you know, call it me being naïve, I just fought through it

Both these examples draw attention to the challenges faced with being a woman in a technical or leadership role in competitive sports, one in the arena trying to make a difference, and the other showing the need for perseverance to even enter the arena and sensing the roadblocks to advancement was gender related.

### Coping skills

Coping with the stressors of work can take many forms affecting mood, feelings, and behavior. The behavioral coping strategies worthy of note these women exhibited clearly in these interviews, include confidence in their abilities, self-belief, and an unrelenting perseverance. Andrea exhibits her confidence in her abilities in the following statement,

I did have an impact on the culture in terms of having high standards setting the standard and expecting nothing lower than that standard. I wanted people to come in and I told them I want you to raise the standard of our operations. Don't lower it, if you want to lower it, get out.

There is no false bravado in this statement, it is a clear expression of the way the training environment was expected to operate. Like Andrea, Teena showed confidence in her abilities in her position, even if the organization took time to understand her role, she was patient, exhibiting a self-belief in her understanding of her role.

I felt I had a good understanding of what I was trying to do, but I would say that my own views and my own ideas definitely were heavily influenced during the first few months and I made a bit of a decision to use the first season as more of a listening tour and more of an opportunity to observe and better understand the cultural dynamics and the people and what not and to slow down the pace of change.

All the participants demonstrated verbally their knowledge and understanding of the positions they held. Indeed, they were selected to participate in this study due to their demonstrated depth of knowledge and experience in their field. However, this may not have been the only factors in their appointment as a first. Their ability to persevere through challenges, their confidence and self-belief in their ability to “get the job done”, illustrated by Ursula's statement and indicative of several of these firsts,

So, I think I was by nature I am somebody who wanted to take on challenges like I'm motivated by challenges. I'm motivated by someone telling me you can't. That can't be done. You can't do that, or women shouldn't do that, or women can't do that. I think that intrinsically motivates me like the fact that there is a barrier makes me want to push the barrier down.

The following quote from Teena in her role as the Vice President of Health and Performance represents so succinctly the overall thoughts of the women in this study,

I would say relentless persistence, certainly, grateful to have had the opportunity to do these jobs, but you know grateful first of all, because somebody's got to be first and we have to move the needle, right?

## Discussion

The purpose of this study was to investigate the experiences of six women who had the distinction of being the first female to occupy their position in a high-level sports organization. Our findings indicate that these women showed character attributes such as leadership qualities that included building and maintaining strong relationships with both male and female colleagues. Of interest is the combination of supportive and non-supportive roles males working in sports organizations have played in the careers of these women. Challenges have been faced and overcome with strong coping skills such as self-belief, confidence, and perseverance. The following discussion highlights their lived experiences.

### Character traits—leadership abilities and relationships

Building relationships has proved advantageous in the development of their careers as firsts. Indeed, five of these women refer solely to male colleagues and mentors who were instrumental in hiring or promoting them into the positions they held. Taylor et al. (7) discussed the need for female mentors as it is difficult to aspire to something you cannot see, sport is lacking female role models. All the participants discussed their skill in networking and maintaining relationships, with both male and female peers, which ultimately was a factor in being appointed to their firsts position (29). Whilst it is purely her own speculation, perhaps Teena was hired because of her commitment to transformational leadership which attempts to challenge a performance team and promote changes within the organization as opposed to transactional leadership which fills a position with the intention of continuing the status quo (30, 31). It can be assumed that transformational innovation calls for not only strong leadership skills but the ability to develop and maintain lasting relationships which are designed to evoke the change (1). Clearly, a similar situation, but working in an organization which is comprised predominately of men, a woman surely must consider nurturing relationships that build trust and can be the hallmark of a success tenure (32).

### Challenges-being female/supportive and non-supportive experiences

Burton (1) stated “Any discussion of women's leadership experiences in sport must include positioning gender as a fundamental aspect of organizational and social processes” (p. 156). Unfortunately, as highlighted earlier, the research literature on women coaches involved in sport has primarily focused on numbers or barriers rather than discussing practices to attract female coaches (7). The findings in this study of “firsts in their field” appears to contradict much of the present literature regarding barriers to obtaining high level position in sport organizations. Seen as high-level performers (not high-level females!) the women interviewed were overwhelmingly encouraged by men who hired them. Could this group be an anomaly because they are all firsts? Possibly so, but interesting to note, this could confirm that these women were fortunate to be supported by male mentors, while others were not so fortunate. This begs the question why them, what do/did they have that others did not? All in this group of firsts were aware of the challenges faced by women in the field as demonstrated by Rocky's comment, “I sure did not want to screw it up” referring to her desire to be successful in her position as she saw her position as paving the way for others. It is important to attempt to ascertain exactly what these women exhibit that earned them the position of a “first”. Perhaps, a proven track record of success either in building a reputation for success, or simply being outstanding at your job, drew attention to these women highlighted by Sue's statement, “I just did what I did and did not know I was opening any doors”. In most of these cases there appears to have been no burning ambition to rise through the ranks to become the first female to perform their jobs. Indeed, it appears that their commitment to their work was recognized and encouraged by both men and women colleagues. Contrast Sue's approach with Teena who had built a strong High-Performance Team in her previous position and felt she had something to contribute, and so was approached regarding a position in the National Basketball Association (NBA) and was appointed based on her knowledge of High-Performance team building. Being a woman seemingly, posed no barrier in these cases.

In this discussion, and against the literature presented in the Introduction, we would be remiss not to address the negative issues these women experienced and witnessed by some comment such as “it was not easy” and “I just fought through it” referring to challenges they encountered when interacting with male members of their organization. Interestingly, during the interview process, none of these women dwelt on these experiences.

### Coping skills—self-belief, perseverance, confidence

There is little doubt that self-belief and confidence are the hallmarks of an individual's ability to accomplish their chosen goal (33). In that respect, the findings of this study are supported by previous research which has focused on expertise as a route to success (34).

The ability to work through challenging times and reach a successful conclusion or persevere to accomplish a successful outcome is exhibited by Ursula in her position as President and now Vice President of the International Weightlifting Federation. She recalls “there were times when I was the only no vote on the board”. She encountered harassment, challenges from board members, threats from the previous President, but she persevered through challenging times to help lead International Weightlifting to a more productive time.

While women will encounter harassment, disrespect, and situations where they are discounted (35), with our participants as a group of firsts, some felt they were and some did not, the ones who did, simply ignored it, or dealt with it and continued on. Our findings reinforce the idea that a pathway for women to be successful in high performance sport is possible, but we quote a statement from most of these women that “it was not easy”. These women lived, worked, and talked of their experiences in the U.S. The single geographical scope is a limitation of this study. It would be interesting to widen the scope of this study and include other parts of the world and explore the experience women sports leaders more broadly. Furthermore, this study involved interviewing firsts who are now all over the age of fifty. These women have discussed their experiences from what could be considered somewhat of a historical viewpoint. Further investigation could deal with younger contemporary leaders in sports organization, and such research may reveal differences in the past vs. present day, what is different now, how far have we come as women in the field, and what has changed.

In summary, our main findings indicate the women who participated in the study demonstrated strong leadership qualities (28) with an ability to foster good relationships: the hallmark of transformational leadership. Research (36) suggests those women maybe more suited to transformational leadership which depends on relationships for success. All these women discussed the important role relationships played in their abilities to gain and maintain their position. Interestingly, male mentors and supporters played a valuable role in gaining and maintaining their position. However, they were acutely aware of potential gender-oriented negativity which can affect the advancement of women working in sport. Although they were aware of the challenge of being a woman, they were resolute: as one of the interviewed women stated, “I just fought through it.” Further, our findings suggest a strong self-belief, confidence in their abilities, and an unshakable perseverance were major influences leading them to be the “firsts in their field.”

## Data Availability

The datasets presented in this article are not readily available because Ethics Commitee will not allow the release of datasets are private communications. Requests to access the datasets should be directed to stoneme@etsu.edu
